# Patient and Health Professional Perceptions of Telemonitoring for Hypertension Management: Qualitative Study

**DOI:** 10.2196/32874

**Published:** 2022-06-10

**Authors:** Juliana Baratta, Cati Brown-Johnson, Nadia Safaeinili, Lisa Goldman Rosas, Latha Palaniappan, Marcy Winget, Megan Mahoney

**Affiliations:** 1 Division of Primary Care and Population Health Stanford School of Medicine Palo Alto, CA United States

**Keywords:** hypertension, remote blood pressure monitoring, precision health, mobile phone

## Abstract

**Background:**

Hypertension is the most prevalent and important risk factor for cardiovascular disease, affecting nearly 50% of the US adult population; however, only 30% of these patients achieve controlled blood pressure (BP). Incorporating strategies into primary care that take into consideration individual patient needs, such as remote BP monitoring, may improve hypertension management.

**Objective:**

From March 2018 to December 2018, Stanford implemented a precision health pilot called Humanwide, which aimed to leverage high-technology and high-touch medicine to tailor individualized care for conditions such as hypertension. We examined multi-stakeholder perceptions of hypertension management in Humanwide to evaluate the program’s acceptability, appropriateness, feasibility, and sustainability.

**Methods:**

We conducted semistructured interviews with 16 patients and 15 health professionals to assess their experiences with hypertension management in Humanwide. We transcribed and analyzed the interviews using a hybrid approach of inductive and deductive analysis to identify common themes around hypertension management and consensus methods to ensure reliability and validity.

**Results:**

A total of 63% (10/16) of the patients and 40% (6/15) of the health professionals mentioned hypertension in the context of Humanwide. These participants reported that remote BP monitoring improved motivation, BP control, and overall clinic efficiency. The health professionals discussed feasibility challenges, including the time needed to analyze BP data and provide individualized feedback, integration of BP data, technological difficulties with the BP cuff, and decreased patient use of remote BP monitoring over time.

**Conclusions:**

Remote BP monitoring for hypertension management in Humanwide was acceptable to patients and health professionals and appropriate for care. Important challenges need to be addressed to improve the feasibility and sustainability of this approach by leveraging team-based care, engaging patients to sustain remote BP monitoring, standardizing electronic medical record integration of BP measurements, and finding more user-friendly BP cuffs.

## Introduction

### Background

Hypertension is the most prevalent and important risk factor for cardiovascular disease, affecting 1 in 4 adults worldwide [1]. According to the Centers for Disease Control and Prevention, in 2014, hypertension was the underlying cause of death of 410,000 Americans, with >1100 deaths each day [2]. Nearly half of US adults are hypertensive, with a blood pressure (BP) >130/80 mm Hg. If left untreated, hypertension increases the risk of heart attack, stroke, kidney disease, and Alzheimer disease [3]. The risk of these adverse consequences can be mitigated through BP reduction by adhering to hypertension treatment, including behavior modification (eg, low-sodium diet and regular exercise) and taking medication, as recommended by the American Heart Association (AHA) guidelines [2].

Adherence to hypertension treatment has been associated with reductions of 35% to 40% in stroke incidence, 20% to 25% in myocardial infarction incidence, and 50% in heart failure incidence [4]. However, only 54% of US patients with hypertension have controlled BP [5]. Challenges to achieving controlled BP include failure to respond to medication, treatment side effects leading to subpar adherence, and lack of engagement in preventive behaviors such as adopting a healthy diet and increasing physical activity [6,7]. Thus, there is a need to advance hypertension management through individualized approaches that engage patients.

Precision health is an emerging approach to patient-centered care [8] that incorporates patients’ variations in genes, environment, and behavioral lifestyle to construct personalized treatment and prevention approaches [9]. From a population health perspective, precision health can improve prevention of heart disease by defining subgroups of patients with hypertension that may benefit from specific therapies [6]. For the individual patient, hypertension management using precision health can enable better targeting of personalized treatment options by identifying high-risk patients or those in early disease stages in the hopes of averting negative outcomes in the future [10]. Previous research has shown that remote BP monitoring combined with health coaching [11] or pharmacist management [12] improves BP control by providing consistent and accurate BP data to both patients and physicians, which is then used to inform the selection of more effective treatment options [13]. Remote BP monitoring has also been shown to empower patients in relation to managing their hypertension [14] and improve the patient-clinician alliance [15].

As a result of the COVID-19 pandemic, there is an even greater clinical need for remote BP monitoring for hypertension management, as reflected by recent policies. On March 20, 2020, the Food and Drug Administration issued an enforcement policy for the expedited use and availability of digital remote monitoring equipment to facilitate patient monitoring during COVID-19 [16]. Although this policy is only to remain in effect during the pandemic, recent uptakes of remote BP monitoring may be sustained owing to support from other policies. For example, in 2019, the National Committee for Quality Assurance updated the hypertension quality measure to allow BP readings to be taken using remote patient monitoring devices and telehealth encounters to satisfy certain components of the quality measure [17]. The use of home BP measurement is also recommended for the ongoing diagnosis and treatment of hypertension in both the 2020 International Society of Hypertension Global Hypertension Practice Guidelines [18] and the 2017 American College of Cardiology and AHA Blood Pressure Guidelines [19].

As a result, remote BP monitoring is becoming increasingly important for hypertension management and, more broadly, in population health programs. Despite recommendations, adoption is precluded by mediators and moderators of remote BP monitoring integration, including the usability of the digital health tools, ease of clinical workflow incorporation, and availability of technical support [20]. Research is still needed to identify the best practices to sustain remote BP monitoring by overcoming barriers on both the patient side (ie, reductions in motivation) and the clinic side, such as the high costs of hardware maintenance and related software for digital health monitoring [21].

### Objectives

Stanford conducted a precision health pilot, Humanwide, to assess the feasibility of embedding a precision health model in a community-based primary care clinic [10]. The goal of Humanwide was to deliver precision health through a combination of “high tech and high touch” care via the use of genetic and pharmacogenomic testing, digital health monitoring, and intensive one-on-one health coaching in the context of team-based primary care [22]. To our knowledge, this is the first implementation of a multipronged precision health delivery model integrated into a primary care clinic. Considering the continuing implementation hurdles that BP management poses and the potential of precision health in this space of patient care, the purpose of this analysis was to formally assess the implementation outcomes of *feasibility*, *acceptability*, *appropriateness*, and *sustainability* [23] of hypertension management via Humanwide and examine multi-stakeholder perceptions of this approach.

## Methods

### Overview

The implementation of Humanwide took place between March 2018 and December 2018. Patient participation in this study was entirely optional. After enrollment in the pilot study, patient information was shared securely with researchers (NS and CBJ), who then contacted patients to determine their interest in participating in the evaluation. Before conducting the interviews, the evaluation team obtained the participants’ verbal consent. The participants were made aware that the interviews would be confidential and in no way affect their care. Interviews and transcripts were only accessed by the external evaluation team and not by Humanwide health professionals. Audio and transcription files were maintained in Health Insurance Portability and Accountability Act–compliant box files. The deidentified aggregate findings were shared with health professionals and Humanwide team members.

### Overview of Humanwide Pilot

Humanwide entailed four components that were added to standard primary care: (1) a baseline wellness visit to assess patient lifestyle, demographics, and socioenvironmental health factors, with follow-up health coaching visits as needed; (2) digital health remote biometric monitoring through the HealthKit app (Apple Inc) [24], including Bluetooth-enabled home scale glucometer, BP cuff for intermittent remote BP readings via the Withings device and app [25], and pedometer; (3) family history assessment and follow-up genetic testing for patients identified from the assessment as at risk for breast cancer, familial hypercholesterolemia, or Lynch syndrome; and (4) pharmacogenomic testing to examine a patient’s likely response to a given class of drugs given their genetic makeup [10]. Implementing these components required care coordination across multiple physicians and specialists, medical assistants (MAs), a pharmacist, a behavioral health practitioner, a registered nurse, and a genetics counselor. The inclusion criteria were (1) adults aged >18 years, (2) seeing a health professional at the pilot study clinic, (3) having a smartphone (to take part in the digital health component), and (4) having time to participate. Health professionals recruited patients that they felt could benefit from Humanwide components, such as medically complex patients managing more than one chronic illness. Attention was given to recruiting diverse patients with respect to age, race and ethnicity, gender, and medical complexities. Primary care health professionals invited 69 patients to participate in Humanwide, and 50 (72%) enrolled. The 19 (28%) patients who declined enrollment reported lack of time as their main reason for nonenrollment.

### Interviews and Data Collection

All patients and health professionals involved with Humanwide were eligible for interviews, and patient recruitment for interviews occurred simultaneously during Humanwide enrollment. Health professionals in the primary clinic and specialists outside of the clinic who were contributing to patient care as part of the pilot were included in the interviews. Patients and health professionals were recruited for interviews via convenience sampling. We tracked roles (ie, primary care physician [PCP], nurse, or pharmacist) to aim for a purposive sample.

We developed a semistructured interview guide to assess the implementation outcomes of *feasibility*, *appropriateness*, and *acceptability* of Humanwide [23]. Interview questions assessed perceptions of each pilot component—genetic testing, pharmacogenomics, digital health, and health coaching—and addressed recommendations for future implementation of Humanwide with respect to hypertension management. As the interviews were semistructured, if a patient mentioned hypertension or remote BP monitoring, the questions were then phrased to assess how the pilot components affected their hypertension management (see Table 1 for a summary of the interview guide questions pertaining to hypertension). A total of 3 researchers trained in qualitative methods (NS, JB, and CBJ) conducted audio-recorded interviews in person in a private conference room or over the phone. To the researchers’ knowledge, no other individuals were present during the interviews. Patient interviews ranged from 17 to 36 minutes (mean 25, SD 6.7 minutes). Health professional interviews ranged from 22 to 60 minutes (mean 45, SD 13.9 minutes). No financial or other compensation was provided for participating in the interviews.

**Table 1:** Summary interview guide for health professionals.

Category	Question
Precision health and hypertension	What was your experience like with hypertension management in Humanwide?
Remote blood pressure monitoring	Which parts of remote blood pressure monitoring worked well? Which parts did not work well? What kind of expectations did receiving blood pressure data place on you?
Genetic testing and pharmacogenomics	Can you tell us if and how genetic or pharmacogenomic testing affected hypertension management?
Health coaching	Can you tell us if and how one-on-one health coaching affected hypertension management?
Implementation feasibility and sustainability	What are your thoughts about the sustainability of hypertension management in Humanwide? What would make this approach more sustainable?

### Data Analysis

We used a hybrid qualitative approach integrating a priori and emergent themes [26]. A priori subjects of interest included hypertension, digital health, pharmacogenomics, genetic testing, and health coaching. Emergent themes were identified via thematic analysis, which involved careful reading and rereading of the transcripts in line with the inductive approach [26]. The analysis involved 3 steps. First, JB read all patient and health professional transcripts using NVivo 11 software (QSR International) and coded them using the a priori subjects of interest [27]. Second, JB extracted emergent themes from all transcripts with input from the full authorship team. Third, emergent themes were assessed in relation to the following implementation science outcomes based on Proctor et al [23]: *acceptability* (satisfaction with various aspects), *appropriateness* (perceived fit), *feasibility* (suitability for everyday use and ability to be carried out considering resources, training, and staff), and *sustainability* (facilitators and barriers to spread). Coding questions and novel emergent codes were discussed during weekly meetings with CBJ, NS, and JB over the course of 4 months. In total, 2 researchers (CBJ and NS) conducted quality checks and verified a final coding schema that included codes for a priori concepts and constructs as well as emergent themes.

### Ethics Approval

This study was given a nonresearch determination and Human Subjects Research Exemption protocol 43279 by the Stanford Institutional Review Board.

## Results

### Participants

Of the 50 patients in Humanwide, 16 (32%) participated in the qualitative evaluation. The interviewed patients were diverse—50% (8/16) were non-White, and 56% (9/16) were women (Table 2)—and representative of the 50 patients enrolled based on race and ethnicity, gender, and age. Patients who explicitly referenced hypertension, remote BP monitoring, or both in their interviews were included in the analysis (10/16, 63%). We interviewed 11 health professionals in the Humanwide pilot clinic, including 9 (82%) PCPs, 1 (9%) pharmacist, and 1 (9%) registered nurse; interviews referencing hypertension or BP monitoring were included in the analysis (6/11, 55%). We also interviewed 4 key informant specialist medical doctors (MDs) involved in the pilot whose practices were outside the primary care clinic. The key informants were a cardiovascular geneticist, a pharmacogenomic specialist, a physician expert in biomedical informatics, and a physician specializing in the management of chronic medical conditions, particularly hypertension (Table 3).

**Table 2:** Patient characteristics (N=16).

Characteristic		Patients, n (%)
**Age (years)**		
	30 to 39	3 (19)
	40 to 49	6 (38)
	50 to 59	5 (31)
	60 to 69	2 (13)
**Race and ethnicity**		
	White	8 (50)
	Asian	5 (31)
	Other	3 (19)
**Gender**		
	Men	7 (44)
	Women	9 (56)

**Table 3:** Health professional characteristics (N=10).

Characteristic			Health professionals, n (%)
**Profession**			
	Primary care physician	4 (40)	
	Specialist medical doctor	4 (40)	
	Pharmacist	1 (10)	
	Nurse	1 (10)	
**Gender**			
	Women	7 (70)	
	Men	3 (30)	

### Emergent Qualitative Themes

Overall, the remote BP monitoring component of Humanwide was the only component that the patients mentioned as contributing to hypertension management. Other components (ie, health coaching, pharmacogenomics, and genetic testing) were not mentioned in conjunction with hypertension management. The participants reported that remote BP monitoring led to mixed increases in patient motivation, enhanced patient-clinician engagement, and improved patient hypertensive management. The participants discussed varied efficiency with remote BP monitoring, and the health professionals were overwhelmed by unfiltered BP data and by providing individualized feedback. The health professionals proposed solutions to these barriers, including managing data through electronic medical record (EMR) settings and leveraging team-based care. Table 4 summarizes the themes that emerged from the analysis along with illustrative quotes from the interviews mapped to implementation outcomes.

**Table 4:** Themes and illustrative examples mapped to implementation outcomes.

Implementation outcome and emergent theme		Illustrative examples
**Acceptability**		
	Increased patient motivation	“I figure if I am measuring my blood pressure on a regular basis and I am noticing that it is high, it should be a mental kick in the head that says ‘Oh hey, I am going to do something about this.’” [Patient 8]
	Increased patient-clinician engagement	“I think the key thing that it does is it builds that relationship. If I have to see you in three months, you’re not going to think about doing your blood pressure. Maintaining, pushing a little more of, ‘How are you doing? What’s going on?’ Just trying to understand that. I think our goal was to try to be more engaged in their lives outside of clinic.” [Health professional 4, PCP^a^]
**Appropriateness**		
	Remote blood pressure monitoring perceivably improves patient hypertensive management	“And also some days I feel like really dizzy and like I am about to faint so then I immediately check my blood pressure to see if it is normal because sometimes I feel like my heart is pounding very fast, so anything that I feel that is not right or something is different, I immediately check to see my blood pressure.” [Patient 5]
	Efficiency with remote blood pressure monitoring	“Just because we were able to access how things are at home. We could see what their blood pressure looks like at home versus in the clinic. It saves a lot of time when you’re meeting with patients cause you have all that information ahead of time.” [Health professional 11, specialist MD^b^]
**Feasibility**		
	Efficiency with remote blood pressure monitoring	“Then I can show them the data, hey, look my blood pressure monitor taken this day is this, taken this day is this. Yeah I like the application itself. The application piece is good. It keeps my historical data.” [Patient 7]
	Technical difficulties with blood pressure cuff	“You have to make sure your bluetooth is on and then you have to make sure everything pairs and sometimes with the blood pressure cuff it will like go through the whole thing where it is squeezing and whatever and then it will be like, oh, error, it did not read.” [Patient 6]
	Time lost	“But it does put that added burden back on me to look through it [BP^c^ readings]. I’m getting five to ten trackers, tracking notices, now every single day. The patients have those tracking information back for me. But if I’m trying to look at everything, which...that’s my goal, then it’s too much.” [Health professional 1, PCP]
**Sustainability**		
	Managing data through EMR^d^ settings	“EPIC has some tools to visualize data in general and it is incorporated in those same views. Just as a normal PC doc would visualize BP data um same basic kind of mechanisms and dashboards. But I think it is an area of active discussion and debate. Like are the tools for health professionals and patients to interact with their data are they as good as they could be and how can we make them more useful.” [Health professional 13, specialist MD]
	Sustaining patient motivation	“I think we’re trying with the digital health, and I think we’ll continue to try. I think, again, it has huge benefit for the people that will do it. I think we need to figure out how to get people to do it, but I think it still has great potential.” [Health professional 5, PCP]
	Need for team-based care	“I think our goal was to try to be more engaged in their lives outside of clinic. That was one of the purposes of these things. How do we do that? Do we need more support doing that? Could an MA^e^ do that?” [Health professional 2, PCP]

^a^PCP: primary care physician.

^b^MD: medical doctor.

^c^BP: blood pressure.

^d^EMR: electronic medical record.

^e^MA: medical assistant.

### Implementation Outcomes

Overall, remote BP monitoring was acceptable and appropriate for hypertension management, whereas other components (ie, health coaching, pharmacogenomics, and genetic testing) were not mentioned as contributing to hypertension care. The patients and health professionals reported some barriers to feasibility and sustainability but recommended solutions to overcome these concerns for future implementation.

#### Acceptability

##### Overview

The use of remote BP monitoring to facilitate hypertension management was acceptable to most patients (13/16, 81%) and health professionals (8/10, 80%) and led to increased patient-health professional engagement; however, acceptance was mixed as not all patients with hypertension used it. On the positive side, the patients enjoyed receiving individualized feedback and treatment guidance from physicians; in some cases, this improved the patients’ motivation to make lifestyle modifications and engage in remote BP monitoring. In contrast, there were several technological glitches with the wireless Bluetooth BP cuff used, and it was thus not deemed an acceptable device in the long term by patients or health professionals.

##### Mixed Patient Motivation

The patients perceived remote BP monitoring with the wireless Bluetooth cuff as one of the main contributors to motivation, although maintenance was difficult for some (3/16, 19%). Patients with hypertension reported an improved desire to make behavioral changes such as implementing a routine exercise regimen. They described how seeing their BP measurements made them more conscientious of their health, which increased their motivation for self-management. The patients also reported an increased sense of accountability to their health professional, which further contributed to motivation:

I mean the attention from the medical staff and the fact that it is there in the app [on my smart phone] is going to make me become more efficient at [blood pressure monitoring].

Patient 7

The patients perceived health professional feedback as the “cue to take action” (patient 3) to adhere to treatment.

Despite increased patient motivation, the health professionals reported that patients did not sustain remote BP monitoring:

The [challenge] of Humanwide is that it probably requires some thought on how to adequately coach patients so that they feel really engaged in the use of wearables.

Health professional 12, specialist MD

A total of 13% (2/15) of the health professionals discussed that, as patients with hypertension controlled their BP, they sometimes stopped using the cuff because they felt less incentivized. In addition, the health professionals mentioned that some patients who needed remote BP monitoring did not participate:

It’s insightful to me that it’s just really hard to get buy-in for that [blood pressure cuff]...For those that are interested, it’s great, but it’s very few.

Health professional 2, PCP

A few (3/15, 20%) health professionals discussed not having the bandwidth to reach out to all patients with hypertension who were less engaged in remote BP monitoring; instead, the health professionals capitalized on patients who were engaged using a “nudge-based approach” (health professional 12), “frequent touches” (health professional 4), and “targeted management plans” (health professional 6).

#### Appropriateness

##### Overview

The health professionals and patients perceived remote BP monitoring to be appropriate based on their experiences of improved hypertension management and patient-health professional engagement, with fewer clinic visits. The health professionals reported that diagnosing patients with masked hypertension with the help of remote BP monitoring allowed patients to incorporate lifestyle changes earlier to achieve BP control. In addition, the health professionals reported titrating the medications of patients with hypertension faster because of remote BP monitoring, potentially leading to moderate improvements in overall clinic efficiency. Most patients with hypertension (15/16, 94%) enjoyed the digital health component and found it helpful to achieve controlled BP because they became more aware of their BP and knowledgeable of how to take part in their treatment.

##### Increased Patient-Clinician Engagement

The use of remote BP monitoring appeared to promote patient-clinician engagement and treatment adherence. The health professionals kept in touch with patients by providing individualized feedback on BP data. A patient mentioned the following:

My doctor called me and noted that my blood pressure was higher than it should be and let me know about that, that I need to take action on it.

Patient 8

The patients with hypertension noted that they really “enjoyed” (patient 3) and “appreciated” (patient 6) receiving feedback from the clinical care team. In addition, the patients perceived this feedback as helpful for achieving their behavioral goals.

The health professionals similarly implied that the increased connection with their patients improved patient understanding of and adherence to their treatment regimen:

We have a tighter relationship. When I reach out, it’s not like I saw them last year. They’ll maybe listen a little bit more. We’ll have a little bit more of a conversation.

Health professional 10, pharmacist

Most health professionals (12/15, 80%) mentioned that receiving the BP data placed an expectation on them to reach out to their patients to provide feedback.

#### Improved Patient Hypertensive Management

The participants perceived improved hypertensive management as a result of remote BP monitoring. The participants reported that remote BP monitoring improved “awareness of what was going on at home” (patient 3), as best described by a patient:

Now that there is an application and a digital record that allows me to be more conscientious, “Oh wow, it has been a week, or it has been 4 days, or it has been 3 days,” and, you know, that is now in my mind being more attentive about checking my blood pressure.

Patient 7

The health professionals mentioned at-home BP data serving as a “checkpoint” (health professional 11) to ensure that patients were well-managed. A total of 20% (3/15) of the health professionals noted that some patients had normal BP in the clinic but elevated BP out of the clinic:

Individuals who never had a diagnosis of hypertension were getting blood pressure measurements in the range that would meet the criteria for hypertension as a diagnosis, and then they were able to...make lifestyle changes that were very tailored to them, that then reduced their blood pressure, and prevented hypertension.

Health professional 6, PCP

Home BP monitoring was coupled with regular measurements in the clinic to help ensure accuracy and precision between home and clinic BP measurements.

#### Feasibility

##### Overview

There were concerns regarding the long-term feasibility of hypertension management in Humanwide because of the following barriers: (1) limitations of the BP cuff, including limited sizing, which may have led to inaccurate results for those with high BMI; (2) technical difficulties because of wireless connectivity issues; (3) time lost sifting through overwhelming amounts of BP measurements in the EMR; and (4) the number of health professionals needed to provide individualized feedback to patients. Unfortunately, the patients and health professionals became frustrated with the BP cuff’s technological issues, which led to drops in engagement with remote BP monitoring throughout the pilot. The health professionals also reported significant time lost reading through patient BP data to identify clinically actionable measurements. Although the health professionals believed that providing individualized feedback was beneficial to patients, they felt it was not sustainable unless they used MAs or pharmacists to share this responsibility.

##### Mixed Efficiency With Remote BP Monitoring

There were mixed reports on the efficiency in managing patients with hypertension as a result of remote BP monitoring. Although some participants felt that remote BP monitoring made it easier to track BP and contributed to increased clinic efficiency, others mentioned technological glitches reducing efficiency and motivation. The patients reported that the wireless Bluetooth feature of the BP cuff saved time and made tracking BP data easy:

Yeah I like the application itself. The application piece is good. It keeps my historical data.

Patient 1

A health professional ratified the value of this wireless Bluetooth technology considering the BP reads auto-populate the patient’s app and medical chart:

I don’t have to call my patient asking them to read back some blood pressures, and conversely I know when they are not recording their blood pressures.

Health professional 13, specialist MD

This example illustrates the potential of remote BP monitoring to improve health professionals’ awareness of patient progress and inform individualized treatment.

The health professionals also discussed that having patient BP data before a clinic visit saved time and made the visit more productive. The health professionals implied that it improved clinic efficiency as it took fewer visits to stabilize patients on their medication:

I think we’re able to titrate them quicker, to make the blood pressure medicine changes quicker than we would having to come into the office every three to six months. So, we get them to goal quicker.

Health professional 2, PCP

Of the 15 health professionals, 9 (60%) corroborated that they became “pretty efficient in terms of the workflow” (health professional 4) since implementing remote BP monitoring.

Although the participants discussed increased efficiency with remote BP monitoring, many expressed concerns over technological glitches with the wireless Bluetooth BP cuff. The patients mentioned difficulties with the BP readings not syncing to their EMR and the cuff not taking measurements:

They are a little glitchy. The blood pressure cuff is probably the easiest [remote monitoring digital health tool] to use but there are several steps involved.

Patient 6

The health professionals echoed this sentiment, reporting that patients did not regularly monitor BP because of glitches with the technology:

They [patients] get frustrated when they put the cuff on, and it doesn’t work. They don’t want to do it.

Health professional 2, PCP

Technological difficulties also affected health professional workflow because the health professionals often had to help patients troubleshoot over the phone or schedule a patient visit:

With the BP cuff, sometimes there were technical issues and they [patients] kind of knew to ask me or I gave them resources to call.

Health professional 11, specialist MD

Indeed, the pilot intake process included a full hour with an MA entirely devoted to troubleshooting digital health devices. The patients mentioned needing to adjust their arm position for the cuff to work, and a patient required an additional office visit for guidance.

#### Sustainability

##### Overview

The health professionals mentioned several concerns related to the sustainability of remote BP monitoring as part of hypertension management. They discussed the negative impacts on the sustainability of remote BP workflows and patient motivation and engagement from feasibility issues: BP cuff technical glitches reducing motivation for patients, health professional time lost sifting through BP measurements, and time constraints limiting health professionals’ individualized feedback to patients. According to patient and health professional interviewees, the patients measured their BP daily, especially during the first half of the intervention period (March 2018 to July 2018), with a gradual drop in frequency of BP measurements in the latter half of the pilot.

The health professionals suggested several solutions to overcome feasibility and sustainability issues, including using a different wireless Bluetooth cuff in the future, providing patients with more information or guidance on how to properly use the cuff, better leveraging team-based care, and enabling measurement of BP data through automated EMR settings. Ideally, guidance for BP cuff use would be accessed outside the clinic through video tutorials or on-demand technological support. EMR settings could be adjusted such that health professionals receive an alert only when a patient’s BP is above a certain cutoff. A final suggestion was to incorporate artificial intelligence (AI)-based BP cuffs that would only surface alarming BP measurements. Overall, a great deal of care coordination, technological improvements, and approaches to sustain remote BP monitoring needs to be addressed to achieve long-term sustainability.

##### Overwhelmed by Unfiltered Data and Providing Individualized Feedback

The health professionals received a deluge of BP data in the EMR from remote monitoring and reported time lost as a result of reviewing these data and following up with patients:

I have to respond to them or do something with the information. But it’s now turning out to be five to ten every single day that I’m getting. And obviously I think it’s useful, which is why I’m doing it. But at some point, I’m gonna say, “This is just too much.”

Health professional 2, PCP

The health professionals reported that providing tailored feedback to patients was appropriate for hypertension management but acknowledged that the current workflow, where health professionals are solely responsible for communication, might not be acceptable in the long term. Conversely, several health professionals implied that daily communication with patients was worth their time even though there were no shortcuts:

You can’t just say, “Do this,” which is fine. I mean, you have to understand that it’s just not going to be a quick [fix]. I mean, it’s that relationship, right? You’re building that relationship.

Health professional 2, PCP

A health professional even felt that daily communication was sustainable:

The frequent touches and the frequent follow-up is very doable.

Health professional 4

### Health Professional Proposed Solutions

#### Managing Data Through EMR Settings

The health professionals managed BP data through EMR settings to reduce extra time spent sifting through BP measurements by setting “overs and unders” (health professional 12) so they would only be notified when a patient’s BP was too high or low. Another strategy included setting the EMR to receive BP data every 2 weeks. The health professionals reported that it was not feasible to check BP measurements in real time. A health professional emphasized advising patients with hypertension to seek emergency help as they normally would if they encountered alarming BP measurements in conjunction with signs and symptoms of a hypertension emergency. If the patients were experiencing high BP measurements in isolation, they were advised to reach out to their PCP for potential medication adjustments or follow-up visits.

A few health professionals (3/15, 20%) also suggested developing graphical displays of BP measurements over time that could be visualized within the EMR to capture trends, outliers, and average BP values:

Graphical displays are the most meaningful for the health professional who is looking at it [BP data]. Most of my colleagues, when we are in clinic, we look at the graphical trend over time and we look at the average.

Health professional 6, PCP

Optimal visualization of BP data via the EMR remains an area of “active discussion and debate” (health professional 13).

#### Need for Team-Based Care

Many health professionals emphasized the importance of team-based care to enable successful hypertension management. A total of 40% (6/15) of the health professionals mentioned using MAs and pharmacists when treating patients with hypertension and the importance of everyone working at the top of their license. To best integrate remote BP monitoring, 13% (2/15) of the health professionals discussed needing to alter the typical patient-health professional model:

If you just apply technology to existing workflows and models you are not necessarily going to have better outcomes. You need to figure out, “What care models do these new technologies enable?” And it is things like centralization and different care team members interacting differently to data.

Health professional 13

The health professionals expressed that not all health care systems will have the resources to implement remote BP monitoring and individualized health professional feedback. However, a health professional discussed the potential for web-based patient management with the help of remote BP monitoring:

We are trying to build on top of that kind of precision health approach with a protocolized team-based strategy for remote patient monitoring and appropriate care referral, with the thought being that most physicians or health professionals may have difficulty seeing their patients more regularly than 3 months just because access is limited.

Health professional 12, specialist MD

The health professionals also mentioned the ability of remote BP monitoring to reduce patient visits and, therefore, increase clinic efficiency.

## Discussion

### Principal Findings

This study explored emergent themes along with facilitators and barriers to hypertension management in the first reported precision health pilot study integrated into a primary care setting. The participants reported that remote BP monitoring led to improvements in patient treatment adherence and lifestyle behavior changes. These accounts are similar to those of previous studies showing improved medication management and adoption of lifestyle changes in patients using remote BP monitoring and health coaching [28,29]. The purpose of this evaluation was to assess the implementation outcomes for the Humanwide precision health pilot to inform future expansion of the intervention.

Most health professionals (9/15, 60%) stated that remote BP measurements are helpful for managing patients with hypertension as they are “actionable” and “are providing a new source of ground truth” (health professional 16, specialist MD). The AHA similarly recommends remote BP monitoring for a more comprehensive view of patients’ BP control [30]. Other benefits of remote BP monitoring coupled with office BP measurements include better prediction of cardiovascular morbidity and mortality, improved patient understanding of hypertension management leading to better treatment adherence, and increased detection of BP variability [31].

The health professionals mentioned difficulty in obtaining patient buy-in to start remote BP monitoring. PCPs from a recent qualitative study suggested patient education sessions to enhance patient engagement by emphasizing how remote BP monitoring can be a source of individual empowerment in clinical care [32]. Another study proposed a model based on the business process management paradigm to empower patients by setting negotiated health goals and providing consistent lines of communication between the patient and their health care team to help trigger initial engagement in remote BP monitoring [33].

Along with the difficulty in encouraging patients to initially engage in remote BP monitoring, there were concerns that motivation was not sustainable in the long term. Some patients reportedly stopped using their cuff near the end of the pilot, potentially because they achieved BP control. However, patients who achieve well-controlled hypertension should continue monitoring their BP on a semiregular basis [30]. Common patient-level moderators of BP control include self-efficacy, self-awareness, and education [34]. Self-monitoring of lifestyle behaviors (eg, diet, physical activity, and sleep) tends to be an effective tool for *changing* behavior but may be less well-suited for *maintaining* behavior as it is challenging to do so in the long term [31]. Although some studies show that remote BP monitoring combined with telehealth counseling could improve adherence to hypertension care [35], most patients (14/16, 88%) did not report engaging in health coaching sessions. We believe that this lack of engagement was potentially due to greater interest in the more novel pilot components of genetic testing, pharmacogenomic testing, and Bluetooth devices. Although the participants did receive genetic and pharmacogenomic testing, the results of these tests did not significantly affect their hypertension management or treatment regime.

Tools to improve long-term engagement in BP monitoring are needed, and future strategies could include a more systematized nudge-based approach in which health professionals regularly provide patients feedback based on their BP measurements. Future work could also explore a combination of remote BP monitoring, health professional feedback, and engaging patients’ social support members (ie, family and friends), much like an intervention currently being studied at Penn Family Care [36].

At the health care system level, physicians had several suggestions to address the barriers that surfaced, including leveraging team-based care to sift through patient BP measurements and buoy individualized feedback to patients, AI-based tools to surface clinically relevant data, and improved BP cuff technology. A recent meta-analysis showed that multicomponent strategies, including team-based care and medication titration by a nonphysician (ie, an MA or pharmacist), were most effective for systolic BP reduction compared with other interventions [37]. A study found that most patients are interested in using AI-based tools in their care [38], which may someday include an AI-based algorithm in development that differentiates clinically relevant BP measurements from outliers and extraneous data [39]. In addition, the AHA recently called for improved BP cuff technology and a set of clear standards on how to use these cuffs, echoing this study’s issue with technical glitches with the BP cuffs used [40]. Bluetooth-enabled BP cuffs can allow clinicians to monitor patterns in patients’ BP data. However, there are several access barriers to consider, including the need for a smartphone app to connect the Bluetooth BP cuff and consistent Wi-Fi, which may not be affordable for all patients or available in rural areas [41]. Future research is needed to explore patients’ and health professionals’ perceptions of Bluetooth-enabled BP cuffs relative to manual BP cuffs for home monitoring. In addition, implementation science research is necessary to determine whether Bluetooth-enabled BP cuffs can be implemented in various settings given their cost and mixed feasibility.

The COVID-19 pandemic has surfaced a clinical need and demand for remote BP monitoring. According to a study published by the AHA, BP control worsened in both men and women at the onset of the COVID-19 pandemic [42]. A recent qualitative study found that PCPs believe that remote BP monitoring can improve hypertension management, but successful implementation requires improving patient acceptance and seamless integration into clinical workflows [32]. We would expect remote BP monitoring to become the gold standard based on this evaluation and previous research. For remote BP monitoring to be accessible and sustainably used, there would need to be improvements in BP data visualization and EMR incorporation [43], Bluetooth technology [44], insurance coverage of Bluetooth BP cuff technology [45], and team-based care [46].

### Strengths and Limitations

Several limitations should be noted for interpreting the study findings, including testing in a single clinic, the small number of patients and health professionals interviewed, and the absence of quantitative data to assess actual patient changes in BP. Of the 50 patients in the Humanwide pilot, 34 (68%) were not interviewed because of our convenience sampling method. This method has known limitations, including lack of generalization, inability to represent subpopulations accurately, and bias toward people who will participate [47]. To account for these biases, we attended to demographics of the sample, making a purposive attempt to include perspectives from a variety of roles for clinicians and determining that our demographic balance for patients would include all groups of interest.

Thematic saturation has been systematically assessed in previous qualitative studies, with determinations that thematic saturation was reached in 2 studies at 12 interviews [48,49]. As previously mentioned, our sample sizes for health professionals and patients were relatively small (n=15 and n=16). On the basis of previous work with saturation and our assessment of no novel themes regarding BP, we found our patient data set to represent thematic saturation. Our clinician data set confirmed the themes from the patient data set. This agreement between data sets is a strong indicator through data triangulation [50] that our findings represent thematic saturation of BP perspectives in this pilot. All patients who mentioned remote BP monitoring or hypertension (10/16, 63%) were included in the data analysis. This study highlights the experiences of multiple stakeholders, including patients and health professionals, to inform the future dissemination of hypertension management in Humanwide and precision health more broadly.

### Conclusions

We found that, of the 4 components of Humanwide (pharmacogenomics, genetic testing, digital health, and health coaching), digital health via remote BP monitoring was reported to be the most impactful for hypertension management from the perspective of both patients and health professionals. Despite the barriers, remote BP monitoring is promising, as reflected by enhanced patient-health professional engagement, perceived improvements in patient care, and increased clinic efficiency. Future recommendations to overcome barriers include the integration of patient BP data into the EMR, automated ways of monitoring and identifying actionable BP measurements, leveraging team-based care to facilitate data monitoring and individualized feedback, and tools to increase sustained patient use.

## Abbreviations

AHA: American Heart Association
AI: artificial intelligence
BP: blood pressure
EMR: electronic medical record
MA: medical assistant
MD: medical doctor
PCP: primary care physician
